# 16p11.2 deletion in patients with paroxysmal kinesigenic dyskinesia but without intellectual disability

**DOI:** 10.1002/brb3.1134

**Published:** 2018-10-11

**Authors:** Wen Li, Yifan Wang, Bin Li, Bin Tang, Hui Sun, Jinxing Lai, Na He, Bingmei Li, Heng Meng, Weiping Liao, Xiaorong Liu

**Affiliations:** ^1^ Institute of Neuroscience the Second Affiliated Hospital of Guangzhou Medical University Guangzhou China; ^2^ Key Laboratory of Neurogenetics and Channelopathies of Guangdong Province the Ministry of Education of China Guangzhou China; ^3^ Department of Neurology, the First Affiliated Hospital and Clinical Neuroscience Institute Jinan University Guangzhou China

**Keywords:** 16p11.2 deletion, copy number variants, paroxysmal kinesigenic dyskinesia, *PRRT2* gene

## Abstract

**Introduction:**

Mutations of the *PRRT2* gene are the most common cause for paroxysmal kinesigenic dyskinesia. However, patients with negative *PRRT2* mutations are not rare. The aim of this study is to determine whether copy number variant of *PRRT2* gene is another potential pathogenic mechanism in the patients with paroxysmal kinesigenic dyskinesia with negative* PRRT2* point and frameshift mutations.

**Methods:**

We screened *PRRT2* copy number variants using the AccuCopy™ method in 29 patients with paroxysmal kinesigenic dyskinesia with negative *PRRT2* point and frameshift mutations. Next‐generation sequencing was used to determine the chromosomal deletion sites in patients with *PRRT2* copy number variants, and to exclude mutations in other known causative genes for paroxysmal kinesigenic dyskinesia.

**Results:**

Two sporadic patients with negative *PRRT2* point and frameshift mutations (6.9%) were identified to have de novo *PRRT2* copy number deletions (591 and 832 Kb deletions located in 16p11.2). The two patients presented with pure paroxysmal kinesigenic dyskinesia and paroxysmal kinesigenic dyskinesia and benign infantile convulsions, respectively. They had normal intelligence and neuropsychiatric development, in contrast to those previously reported with 16p11.2 deletions complicated with neuropsychiatric disorders. No correlation between the deletion ranges and phenotypic variations was found.

**Conclusion:**

16p11.2 deletions play causative roles in paroxysmal kinesigenic dyskinesia, especially for sporadic cases. Our findings extend the phenotype of 16p11.2 deletions to pure paroxysmal kinesigenic dyskinesia. Screening for 16p11.2 deletions should thus be included in genetic evaluations for patients with paroxysmal kinesigenic dyskinesia.

## 1 INTRODUCTION

Paroxysmal kinesigenic dyskinesia (PKD) is an autosomal dominant inheritance disorder characterized by paroxysmal involuntary dystonic, choreoathetoid, and ballistic attacks triggered by sudden movements (Jankovic & Demirkiran,^2002^). It has been demonstrated to be associated with mutations in several genes, including *PRRT2* (proline‐rich region transmembrane protein‐2) (Chen et al., 2011), * SLC2A1* (solute carrier family 2, member 1), *MR‐1* (myofibrillogenesis regulator 1), *CLCN1* (chloride voltage‐gated channel 1) (Wang, Li, Liu, Wen, & Wu, 2016), *SCN8A* (sodium voltage‐gated channel alpha subunit 8) (Chen et al., 2015), and* ADCY5* (adenylyl cyclase 5) (Gardella et al., 2016). *PRRT2* is the most common known causative gene for PKD. Point and frameshift mutations in *PRRT2* account for 20.8%–48.4% of cases with PKD (Huang et al., 2015; Liu et al., 2013; Youn et al., 2014). Clinically, PKD cases with negative genetic findings are not rare, especially the sporadic cases (Wang et al., 2016). Since the majority of *PRRT2* mutations are truncating mutations that lead to loss of function or haploinsufficiency of *PRRT2,* copy number deletions of this gene are suspected to cause PKD.

In the present study, we examined copy number variants (CNVs) of *PRRT2* in patients with PKD with negative point and frameshift mutations in *PRRT2* using the AccuCopy™ method. Further, next‐generation sequencing (NGS) was performed to determine the range of deletions and exclude abnormalities in other known causative genes for PKD in patients with *PRRT2* CNVs.

## 2 METHODS

### 2.1 Participants

Twenty‐nine PKD patients with negative *PRRT2* point and frameshift mutations, 13 PKD patients with *PRRT2* point or frameshift mutations, and 50 healthy adults were recruited from the Second Affiliated Hospital of Guangzhou Medical University between 2004 and 2016. PKD was diagnosed according to the criteria described by Bruno et al. (2004). The clinical data were collected, including semiology and evolution of the disorder, family history, and physical examination results. Video electroencephalography (VEEG) monitoring and brain magnetic resonance imaging were performed to exclude epilepsy and other symptomatic movement disorders.

The ethics committee of the hospital approved the study protocol, and written informed consent was obtained from all participants or their parents.

### 2.2 Detection of CNVs of *PRRT2* using the AccuCopy™ method

Blood samples were obtained from the probands, their parents, and other family members when available. Genomic DNA was extracted from peripheral blood using a QuickGene DNA whole blood kit L (Fujifilm, Tokyo, Japan). Mutations in known causative genes for PKD were screened using direct Sanger sequencing or a specific NGS gene panel designed for genes associated with epilepsy and paroxysmal movement disorders. In order to rapidly screen the CNV of *PRRT2* in a large number of patients with a limited amount of sample DNA, an AccuCopy™ multicopy number detection method (Genesky Bio‐Tech Co., Ltd., Shanghai, China) was used. Twenty‐nine patients with negative *PRRT2* point and frameshift mutation (P1‐P29) were screened for *PRRT2* CNVs. Thirteen patients with *PRRT2* missense or truncation mutations (PC1‐PC13) and 50 healthy adults (C1‐C50) were also screened as positive controls and healthy controls, respectively.

The basic principle of AccuCopy™ multicopy number detection has been described previously (Du et al., 2012). Five probes were designed to cover the region spanning the 5′‐UTR to 3′‐UTR untranslated region of *PRRT2*. The probe primers are listed in the Supporting Information Table S1. Three reference genes (*POP1*,* RPP14*, and* TBX15*) were utilized for normalization. AccuCopy™ was performed according to the manufacturer’s instructions. Raw data were analyzed using GeneMapper 4.0. The sample/competitive peak ratio for each target fragment was first normalized three times to three reference genes and then averaged. The copy number of each target fragment was calculated using the average sample/competitive ratio. Parental DNA from the patients with *PRRT2* CNVs was used to determine the origins of the gene variants.

### 2.3 Whole genome CNV analysis using high‐throughput NGS in patients with *PRRT2* CNVs

In order to determine the locations of the deletions, high‐throughput whole genome NGS was carried out in patients with CNVs of *PRRT2* by MyGenostics Inc. (Beijing, China). Qualified genomic DNA samples were randomly fragmented into fragments of 100–700 bp and adapters were ligated to both ends of the fragments. The DNA strands were separated into single strands. Strict quality control testing using Nanodrop 2000 (Thermo Fisher Scientific, DE) and quantitative polymerase chain reaction were performed during the entire process of library construction. Each captured library was loaded onto an Illumina HiSeq X10 platform to perform high‐throughput sequencing using paired‐end 150 base‐pair reads. The bioinformatics analysis was performed to utilize sequencing data generated from the complete genomics sequencing platform. The base‐calling software received data from the imager after each reaction cycle to form raw read data. BWA‐SW was used to perform the alignment. Regions of the genome deemed likely to differ from the reference genome were identified using the alignment data. All detected CNVs were compared to known CNVs in publicly available databases, including OMIM (https://omim.org/, RRID: http://scicrunch.org/resolver/SCR_006437), GeneReviews (https://www.ncbi.nlm.nih.gov/pubmed/20301295, RRID: http://scicrunch.org/resolver/SCR_006560), Decipher (https://decipher.sanger.ac.uk/, RRID: http://scicrunch.org/resolver/SCR_006552), ClinVar (https://www.clinicalgenome.org/data-sharing/clinvar/, RRID: http://scicrunch.org/resolver/SCR_006169), and DGV (https://dgv.tcag.ca/dgv/app/home, RRID: http://scicrunch.org/resolver/SCR_007000).

### 2.4 A literature review for PKD patients with 16p11.2 deletions

To explore the genotype‐phenotype association, 16p11.2 deletions with the phenotype of PKD and related phenotypes were systematically retrieved from the PubMed database till December 2017.

## 3 RESULTS

### 3.1 CNVs of *PRRT2*

**Figure 1:**
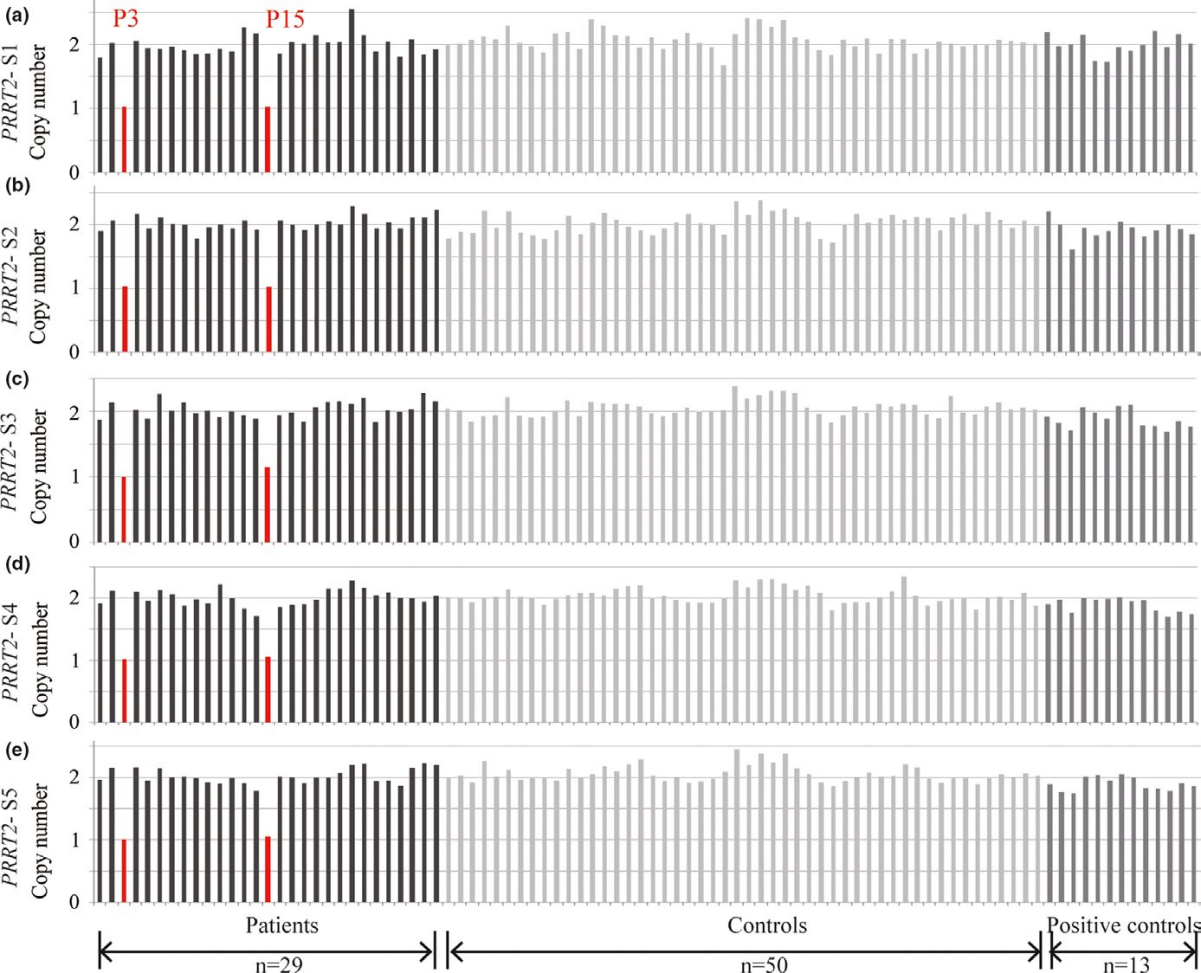
Copy number deletions of *PRRT2* in two patients with PKD detected using AccuCopy™. The copy number of *PRRT2* was determined based on the copy numbers of five target segments (*PRRT2* S1–S5). One copy deletions of segment S1–S5 were identified in patients P3 and P15, indexed by red bars. Black bars represent PKD patients with negative *PRRT2* point and frameshift mutations (P1–P29), light gray bars represent healthy controls (C1–C50), and dark gray bars represent positive controls (PC1–PC13)

*PRRT2* CNVs were analyzed in 29 patients with negative point and frameshift mutations, 13 patients with point and frameshift mutations, and 50 healthy controls. Reduced amplification of target segments (*PRRT2* S1–S5) was found in two (6.9%) patients with negative *PRRT2* point and frameshift mutations (patients P3 and P15), implying that one copy of the whole *PRRT2* gene was deleted. No copy number deletion or duplication of *PRRT2* was found in the rest of the subjects (Figure 1).

**Figure 2:**
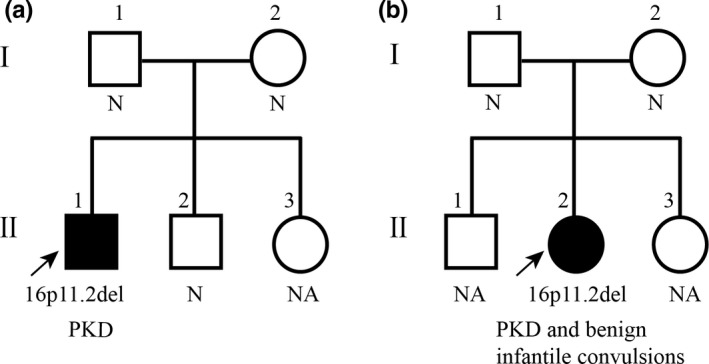
The pedigree of the patients with 16p11.2 deletions. (a) Pedigree of patient P3 who presented with PKD. (b) Pedigree of patient P15 who presented with PKD and benign infantile convulsions. *N* indicates normal copy numbers. NA indicates not available

No copy number deletion was found in the parents of patients P3 and P15 (Figure 2), indicating that the deletions in the two patients were de novo.

**Figure 3:**
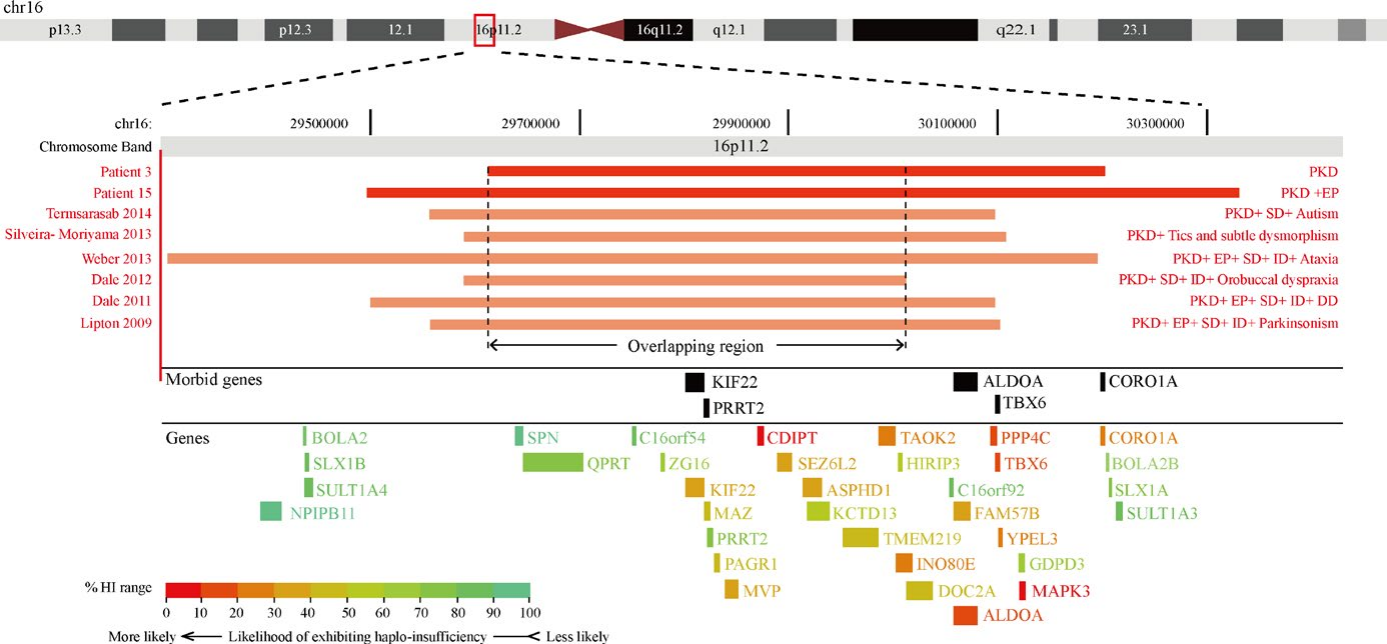
Genomic positions of the deletions and phenotypes of the PKD patients with 16p11.2 deletions. Genomic positions of the 16p11.2 deletions in patients with PKD are shown using red bars. Bright red bars indicate the 16p11.2 deletions in the present study. Light red bars indicate the deletions reported in the literature. Their corresponding phenotypes are listed on the right. The involved genes are produced using the Ensemble Genome Browser (https://grch37.ensembl.org/Homo_sapiens/Location) and listed below the bars. The morbid genes are indexed using black fonts. The haploinsufficiency scores of the involved genes are produced using the DECIPHER database (https://decipher.sanger.ac.uk/) and indexed by colored fonts. DD: developmental delay; EP: epilepsy; ID: intellectual disability; PKD: paroxysmal kinesigenic dyskinesia; SD: speech delay

The ranges of the deletions in patients P3 and P15 were further investigated using NGS. Patient P3 had a 591 kb deletion in chromosome 16 [arr16p11.2 (29615090–30207087) × 1] (Figure 3), which encompassed 37 genes and transcripts, including five Mendelian disease genes (*PRRT2*,* KIF22*,* ALDOA*,* TBX6*, and *CORO1A*). This patient also had a 137 kb deletion in chromosome 2 [arr2p13.2 (72751634–72888731) × 1], which encompassed a part of the *EXOC6B* gene. Patient P15 only had an 832 kb deletion on chromosome 16 [arr16p11.2 (29494072–30326832) × 1], which encompassed 45 genes and transcripts, including the five Mendelian disease genes described above (Figure 3). Of the affected genes, *KIF22* and *TBX6* are not expressed in the brain; thus, their pathogenic roles for PKD were excluded. *CORO1A* is associated with immunodeficiency 8, *ALDOA* is associated with glycogen storage disease 12 and ataxia‐telangiectasia‐like disorder 2, and* EXOC6B* is associated with intellectual disability and developmental delay. No evidence currently suggests that these genes are related to PKD. *PRRT2* is the only known causative gene for PKD in this region.

No mutations in other known causative genes for PKD (including *MR‐1*,* SLC2A1*,* CLCN1*,* SCN8A,* and* ADCY5*) were found in patients P3 and P15 using the NGS gene panel.

### 3.2 Clinical features of the two patients with CNVs of* PRRT2*

Patient P3 was a 17‐year‐old male with normal development and negative family history of PKD and epilepsy. He had his first dystonic attack at 11 years of age. The attack manifested as twitching in the upper limbs and facial muscles when suddenly standing up, accompanied by clasping of all five fingers without loss of consciousness. This lasted for more than 10 s. Since then, the attacks have occurred about 3–4 times per month and are usually exacerbated by sleep deprivation. The patient had no history of febrile seizures or epileptic attacks. Normal VEEG was recorded at the age of eleven. The patient was diagnosed with PKD. The attacks did not respond to valproate acid (VPA; 15 mg kg^−1^ day^−1^) initially, but were controlled by carbamazepine (0.3 g/day) later. The patient had normal intelligence and graduated from a normal senior middle school with average performance. No language disability and autistic features were found (Figure 2a).

Patient P15 was a 22‐year‐old female with negative family history of febrile seizures, epilepsy, and paroxysmal dyskinesia. The patient was normally delivered. Her early childhood development was normal. She presented with epileptic seizures beginning at the age of 8 months with a frequency interval of 2–3 days. She became seizure free for 5 years while being treated with VPA (20 mg kg^−1^ day^−1^), which was withdrawn later. Since the age of 13 years, she presented with uncontrollable choreoathetoid movements of the extremities with awareness when she suddenly changed positions during the daytime. The attacks usually lasted for 2–3 s with a frequency of 6–8 times per month. Her body weight was gradually increased. The body mass index increased from 19.5 at age 12–29.3 at age 22. Neurological examination findings were normal. No speech abnormalities or autistic features were observed. The intelligence test is normal by Wechsler Adult Intelligence Scale at the age of 20 years (IQ = 96). VEEG monitoring showed irregular medium voltage slowing in the right temporal region. No epileptiform discharge was recorded. The patient was diagnosed with PKD and benign infantile convulsions. The PKD attacks were controlled by oxcarbazepine (450 mg/day; Figure 2b).

### 3.3 Phenotypes and genotypes in PKD patients with 16p11.2 deletions

To date, more than 400 patients with 16p11.2 deletions have been reported. Only six of these cases have been reported to have PKD (Dale, Grattan‐Smith, Fung, & Peters, 2011; Dale, Grattan‐Smith, Nicholson, & Peters, 2012; Lipton & Rivkin, 2009; Silveira‐Moriyama et al., 2013; Termsarasab et al., 2014; Weber, Kohler, Hahn, Neubauer, & Muller, 2013). The deletion ranges and phenotypes of these patients with PKD are summarized in Figure 3. Speech delay was found in five patients, intellectual disability was presented in four, and epilepsy was found in three. Autism, orobuccal dyspraxia, developmental delay, ataxia, parkinsonism, tics, and dysmorphism were observed in one patient each. In contrast, the two patients in this study presented with pure PKD or PKD and benign infantile convulsions, but without any other psychological or neurological symptoms. The ranges of the deletions in the eight patients with PKD covered a large region of 903,843 bp (from 29303244 to 30207087). This range includes 34 genes encoding proteins, seven RNA genes, and four pseudogenes. The overlapping range is a 398,398 bp segment from 29615090 to 30013488 and contains 18 encoding genes. Of these genes, only two are known causative ones—*PRRT2* and *KIF22*. *PRRT2* is the gene responsible for the phenotype of PKD. In the range of deletions, no specific gene was found to explain the phenotypes of speech delay and intellectual disability.

## 4 DISCUSSION

Paroxysmal kinesigenic dyskinesia is an autosomal chromosome dominant heredity disorder with obvious heterogeneity of phenotypes and genotypes. *PRRT2* is the most common causative gene for PKD. Mutations of *PRRT2*, including point mutations and frameshift mutations, explain approximately less than one‐half of PKD cases (Huang et al., 2015), suggesting the presence of additional molecular pathogenic mechanisms. In this study, *PRRT2* CNVs were identified in two patients with PKD, indicating that copy number deletion of *PRRT2* is also a potential pathogenic factor for the disease.

In previous studies, point and frameshift mutations in *PRRT2* were identified in 61.5%–100% of familial cases of PKD (Huang et al., 2015; Liu et al., 2013). Even in sporadic cases, the majority point and frameshift mutations of *PRRT2* were inherited from unaffected parents (Ebrahimi‐Fakhari, Saffari, Westenberger, & Klein, 2015). In contrast, the two mutations in this study were de novo. It indicates that screening for *PRRT2* CNV is necessary in patients with PKD, especially in sporadic cases.

In this study, the deletions in the two patients with PKD were beyond the range of the *PRRT2* gene (chr16: 29812087–29815881) and were similar to the range of mutations in chromosome 16p11.2 deletion syndrome. Chromosome 16p11.2 deletion syndrome is a genetic disorder associated with multiple system abnormalities, including intellectual impairment, developmental disorders, psychical and psychological disease, motor hypotonia, seizures, obesity, immune deficiency, syringomyelia, hearing loss, and cardiac defects (Yang et al., 2015). It is the second common microdeletion disorder (Kaminsky et al., 2011), occurring in approximately 1 of 10,000 individuals (Weiss et al., 2008) and 0.4%–0.7% of patients with unexplained intellectual disability.(Kaminsky et al., 2011). Language‐related disorders and intellectual disability are extremely common in patients with 16p11.2 deletion syndrome (Duyzend & Eichler, 2015; Hanson et al., 2015; Olson et al., 2014). Psychiatric and developmental disorders are found in >90% of carriers (Celine & Antony, 2014). Autistic spectrum disorder is detected in about 18%–25% of deletion carriers (Duyzend & Eichler, 2015). However, PKD has only been reported in six patients with 16p11.2 deletions, and these cases were simultaneously complicated with other neuropsychiatric abnormalities, including intellectual disability, speech delay, autism, and dysmorphism. In contrast, the two patients in the present study only presented with PKD or PKD and mild epilepsy, which indicates that pure PKD or relatively pure PKD can be a phenotype of 16p11.2 deletion syndrome.

Since 16p11.2 deletion carriers present with obvious phenotypic heterogeneity, it needs to be determined whether the deleted ranges are correlated to the phenotypes. In previous studies, a 593‐kb deletion at map position B29.5–B30.1 Mb in the proximal region of 16p11.2 was defined as a typical 16p11.2 deletion commonly associated with symptoms of brain developmental abnormalities, while a 220‐kb deletion at map position B28.74–B28.95 Mb in the distal region was defined as an atypical 16p11.2 deletion featured by severe obesity due to the involvement of the* SH2B1* gene, which plays a role in leptin and insulin signaling. PKD patients with *PRRT2* CNVs all had the proximal 16p11.2 deletions (Dale et al., 2011, 2012 ; Lipton & Rivkin, 2009; Silveira‐Moriyama et al., 2013; Termsarasab et al., 2014; Weber et al., 2013). The overlapping region of the deletions contains two known causative genes—*PRRT2* and *KIF22*. *KIF22* is not expressed in the human brain and is associated with spondyloepimetaphyseal dysplasia with multiple dislocations. Therefore, *PRRT2* is the only responsible gene for the symptoms of PKD in this region. Beyond the overlapping region, three other known causative genes are affected by deletions—*TBX6*,* ALDOA*, and *CORO1A* (Figure 3). *TBX6* is not expressed in the brain; and no evidence indicates that* ALDOA* and *CORO1A* are related to neuropsychiatric disorders. Thus, these three deleted genes do not explain the symptom variety seen in the central nervous system in such patients. Patient P3 also had a deletion in chromosome 2. This deletion included a part of the gene *EXOC6B*. Although this gene is associated with intellectual disability and developmental delay (Fruhmesser et al., 2013), patient P3 had clinically normal intelligence and development. Taking together, the molecular basis for the phenotypic variety in PKD patients with 16p11.2 deletions is unknown.

In previous studies, the majority of patients with 16p11.2 deletions had a relatively poor prognosis in intellectual disability, autism, speech delay, and obesity. In contrast, the present study showed that patients with 16p11.2 deletions may present with pure PKD, which had a good response to low‐dose carbamazepine or oxcarbazepine. This finding should be considered in prognostic prediction and genetic counseling for PKD patients. One of the limitations of this study is the limited sample size. Further studies with large cohorts and the functional studies for each involved gene are required to determine the mechanisms underlying the phenotypic diversity of 16p11.2 deletions.

In conclusion, this study identified two de novo 16p11.2 deletions involving *PRRT2* in patients with PKD but without intellectual disability, extending the phenotype of 16p11.2 deletions to typical PKD. Screening for 16p11.2 deletions should be prescribed for patients with PKD, particularly in sporadic cases, although it is not common in the patients with PKD. Further research is required to determine the mechanisms underlying the phenotypic diversity of 16p11.2 deletions.

## CONFLICT OF INTEREST

None declared.
