# Supplementary material for: 16p11.2 deletion in patients with paroxysmal kinesigenic dyskinesia but without intellectual disability
Source: Brain Behav. 2018 Oct 11;8(11):e01134. doi: 10.1002/brb3.1134 (PMC6236233; doi:10.1002/brb3.1134)
Supplement: Supplementary file 1 [file BRB3-8-e01134-s001.docx]

**Table S1** The primers of *PRRT2* gene for multiplex competitive amplification by the AccuCopy^TM^ method

| **Segments** | **Forward Primers** | **Reverse Primers** |
| --- | --- | --- |
| *PRRT2*-S1 | cccaggggcagccttactg | caagttccaggtctctggggaa |
| *PRRT2*-S2 | ccggcgtcgaggtgagac | aatgcgggtgtctggaggg |
| *PRRT2*-S3 | agaaacagcccaggccacag | aagtgagcaaagaggccactgc |
| *PRRT2*-S4 | gtgggggctagattcattcacg | agatgatgggggaggtttgttg |
| *PRRT2*-S5 | CTCCTGACCCCGGCTATGTG | tcaacttaggcggtgagtggg |
